# The CHESS Protocol: A Mixed-Methods Evaluation of an HPV Screening Intervention for Women Living With HIV in Nigeria

**DOI:** 10.3389/ijph.2025.1608716

**Published:** 2025-08-14

**Authors:** Olabanjo Ogunsola, Laura M. Gaydos, Oluseye Ajayi, Maria Dieci, Nadi Kaonga, Olutosin Awolude, Priscilla Ezemelue, Tyree Staple, Kabiru Salami, Ifeoma Idigbe, Oliver Ezechi, Lisa Flowers

**Affiliations:** ^1^ APIN Public Health Initiatives, Abuja, Nigeria; ^2^ Department of Health Policy and Management, Emory University, Atlanta, GA, United States; ^3^ Department of Gynecology and Obstetrics, Emory University, Atlanta, GA, United States; ^4^ Obstetrics and Gynecology Department, College of Medicine, University of Ibadan, Ibadan, Nigeria; ^5^ Clinical Sciences Department, Nigerian Institute of Medical Research, Lagos, Nigeria; ^6^ Department of Sociology, Medical Sociology, University of Ibadan, Ibadan, Nigeria

**Keywords:** implementation science, cervical cancer, HPV, women living with HIV, lay-health, self-collection

## Abstract

**Objectives:**

In this protocol, we describe a planned intervention to adapt the Mother Mentor (MoMent) peer support program for women living with HIV (WLWH). WLWH face a six-fold higher risk of cervical cancer, yet screening and treatment rates remain low in Nigeria.

**Methods:**

Using an implementation science approach, we will engage key stakeholders—including ministries of health, NACA, professional bodies, WLWH, Mentor Mothers, healthcare providers, and development partners (e.g., WHO, US CDC, USAID)—through deliberative democracy to adapt and expand MoMent for home-based HPV screening and follow-up treatment. We will pilot the adapted MoMent HIV+HCC program with 1,500 women in 15 health facilities across Nigeria’s five geopolitical zones. The RE-AIM framework will guide evaluation of reach, adoption, fidelity, effectiveness, and sustainability.

**Results:**

This study is designed to contribute to WHO’s global strategy to eliminate cervical cancer by improving access to home-based screening and care in low-resource settings.

**Conclusion:**

Findings will inform national HPV prevention efforts and may drive broader integration into Nigeria’s cervical cancer program.

**Clinical Trial Registration:**

Clinicaltrials.gov, identifier NCT06751030.

## Introduction

Cervical cancer (CC) is the fourth most common cancer in women globally, with 660,000 new cases and 250,000 deaths in 2022 [1]. Africa hosts 19 of the top 20 countries with the highest incidence [1]. Women living with HIV (WLWH) are at six times higher risk of developing CC [2, 3], and it remains the most common AIDS-defining malignancy [4]. Limited access to screening and treatment accounts for 90% of CC deaths globally [5].

Despite improvements in HIV care, CC risk among WLWH in Nigeria remains unchanged over the last two decades [1, 2]. In Nigeria, CC is the second most common cancer in women, with over 12,000 new cases and nearly 8,000 deaths per year. The government has recently prioritized CC control, initiating HPV vaccination for girls aged 9–14 [6, 7]. However, these efforts do not address the screening needs of adult WLWH.

Cytology-based screening methods like Pap smears are impractical in low and middle income countries (LMICs) due to cost and infrastructure demands [8–10]. Less resource–intensive screening approaches have shown success in detecting and preventing disease [3]. These include visual inspection with acetic acid/Lugol’s iodine (VIA/VILI) [4] and self-collection (SC) tests targeting HPV, the causative agent of CC [5, 6, 11].

HPV SC requires less infrastructure than cytology-based methods, improving accessibility. In sub-Saharan Africa (SSA), 60%–75% of women who develop CC live in rural areas without early screening and treatment facilities [7–9]. Programs delivering services closer to these areas could dramatically expand access and reach underserved populations. HPV self-testing also reduces loss to follow-up common in cytology-based programs. SC samples are suitable for HPV detection [10] and have comparable sensitivity and specificity to provider-collected samples [12].

Nigeria’s MoMent peer-support program, originally designed to improve HIV care, pairs experienced WLWH (who become Mentor Mothers, MMs) with newly diagnosed peers. MoMent significantly improved ART adherence and viral suppression [13] and now operates nationally. Peer support programs like MoMent have positively influenced health-seeking behaviors and care utilization in low-resource settings [14–17], making it an ideal model for a screening intervention. The CHESS (Community, Home-based Education, Screening Services) study builds on MoMent by integrating HPV self-screening into community HIV care.

### Study Aims

1. Use a stakeholder deliberation (SD) conference methodology to adapt the MoMent program to promote home-based HPV cervical cancer screening (HCC) and follow-up treatment for HIV positive women.
2. Implement the MoMent HIV+HCC cervical cancer screening program and assess program reach, effectiveness, adoption, and fidelity.
3. Conduct post-implementation process evaluation of barriers and enablers to program maintenance.

To date, we have completed Specific Aim 1, including the deliberative democracy process, adapting the intervention, and training study personnel for implementation. We have also initiated a soft roll-out of the intervention in Aim 2. The remainder of this protocol outlines details for Specific Aims 2-3.

## Methods

### Overview

This implementation science study evaluates an adapted peer-support/lay-health intervention to increase HPV testing among WLWH and examines implementation, clinical outcomes, and sustainability metrics.

Our implementation process for the adapted MoMent HIV+HCC intervention involves: 1) facilitating SD conferences to collect input on how to adapt the MoMent program for CC control activities; 2) creating and implementing the HIV+ HCC intervention for the MoMent program; 3) measuring reach, adoption and implementation metrics at 6-month after intervention commencement; and 4) measuring intervention maintenance 18 months after intervention commencement. At the time of submitting this protocol, SD conferences were held and the intervention is adapted. We will conduct intervention activities through connections with clinics and existing MoMent programs across 15 clinics in eight Nigerian states.

### Conceptual Frameworks

Our study combines two implementation science frameworks: the Consolidated Framework for Implementation Research (CFIR) and Reach Effectiveness Adoption Implementation and Maintenance (RE-AIM). CFIR considers contextual factors influencing the adaptation and implementation of evidence-based interventions. Although widely used in high-income countries, CFIR’s use in LMICs benefits from accounting for common clinic and system structures. Means et al. proposed adding “characteristics of systems” (e.g., external agent funding priorities, policy alignment, perceived sustainability) and additional “intervention characteristics” (e.g., team collective efficacy) to CFIR for LMICs [18]. We apply CFIR in Aim 1 for formative work to adapt MoMent to CC screening. RE-AIM addresses the slow, inequitable translation of scientific advances into practice [19], has been used in LMICs [20–23], and is typically applied at both organizational and individual levels. We use RE-AIM in Aims 2 and 3 to evaluate implementation of the HIV+HCC-adapted MoMent program overall and by state (see Table 1).

**TABLE 1:** RE-AIM evaluation elements (CHESS: Community, Home-based Education, Screening Services, Nigeria, 2025).

RE-AIM elements	Descriptions (total and by state)	Numerators	Denominators	Data source	Aim	Timing
Reach	Proportion of WLWH who had direct contact with MM at 6 months	Number WLWH who have contact with MM	All WLWH (ages 25–50) who could have been reached	MM logs	2, 3	6 months, 18 months
	Proportion of WLWH identified with hrHPV types (16/18/45/other) at 6 months	Number of WLWH who are HPV 16/18/45/other positive	All WLWH (ages 25–50) who return SC sample	Medical records	2, 3	6 months, 18 months
Adoption	Proportion of clinics in which >70% of MMs have completed peer training at 6 months	Number of MMs who complete training	All MMs hired for study	Training reports	2	6 months
	Proportion of WLWH who return SC sample at 6 & 18 months	Number of WLWH who return SC sample	Number of WLWH who had direct contact with MM	Medical records/MM logs	2, 3	6 months, 18 months
Implementation	Ratio of MMs to WLWH (optimal = 1:50) at 6 months	Number of MMs completing training	Number of eligible WLWH being treated at clinics	Training reports/Medical records	2	6 months
	Proportion of MMs who attained at least a “competent” score on their performance review at 6 & 18 months	Number of MMs who attained a “competent” score on performance review	All MMs who have seen at least 12 weeks of service at 6-month follow-up	Performance review	2, 3	6 months, 18 months
	Proportion of patients treated within 2 weeks of SC sample received at 6 months	Number of WLWH treated within 2 weeks of SC sample received at clinic	Number of WLWH who test positive for HPV 16/18/45	Medical records review	2	6 months
	Fidelity to intervention as planned – proportion of complete MM checklists completed by MMs at 6 months	Number of checklists showing 4 of 6 steps completed for each MM encounter	All MMs who have seen at least 12 weeks of service at 6-month follow-up	MM logs/Employment records	2, 3	6 months, 18 months
Maintenance	Proportion of clinics that maintain target ratio of patient to MM maintained at 18 months	Number of MMs employed (retained or newly recruited/trained)	Number of eligible WLWH being treated at clinics	Employment records/Medical records	3	18 months
	Proportion of clinics conducting ongoing MM refresher trainings at 18 months	Number of annual trainings conducted in each clinic	Number of clinics	Training records	3	18 months
	Proportion of clinics sustaining optimal test to treatment window at 18 months	Number of clinics with optimal test to treatment window	Number of clinics	Medical records review	3	18 months

Aim 1 Use SD methodology to adapt the MoMent program to promote home-based HPV cervical cancer screening (HCC) and follow-up treatment for WLWH.

Aim 1 comprised the formative work that is the foundation for the CHESS study. We focused the adaptation of the MoMent intervention for CC screening on constructs of the CFIR framework deemed by Means and colleagues to be especially relevant to LMIC settings [24]. SD approaches offer a way to engage communities in adoption and expansion of programs such as CC screening [13, 25–30]. Stakeholders include policymakers, clinic administrators, clinicians, HIV peer-support leaders and WLWH. We employed an innovative two-day, SD conference in which participants viewed neutral informational briefings and gained insights into competing advantages and disadvantages related to CC screening approaches to inform their perspectives. Stakeholders then deliberated and reached consensus on feasible adaptation strategies.

Qualitative data collection included deliberation session recordings, transcripts and participant generated pro-con lists of considerations, created during the deliberation. We also held pre- and post-deliberation surveys to assess changes in participant knowledge and attitudes related to CC prevention for WLWH.

Simultaneous to the community SD, we held a one-day expert stakeholder meeting for high-level Nigerian decision makers, including federal stakeholders (e.g., Ministry of Health, National Cancer Control Programme, National Agency for the Control of AIDS), development agencies and implementing partners (e.g., World Health Organization, Centers for Disease Control and Prevention, and USAID). This expert session introduced decision makers to the CHESS study, shared preliminary planning and the outcomes of the SD community process, and solicited feedback on the study plan. This meeting also allowed for buy-in from expert stakeholders, several of whom were asked to sit on the study’s technical advisory group.

Findings from the SD conference were used to adapt the CHESS intervention based on the considerations raised by community deliberators and expert stakeholders. Two key changes to the study protocol were made as a result:

Major Change 1: HPV education and screening were moved from home to community-based locations.

Major Change 2: High-risk HPV results were determined better returned by MMs rather than by nurses to maintain the relationship with patients and help facilitate next treatment steps for patients with high-risk results.

Aim 2 Implementation and Assessment of Reach, effectiveness, adoption, and fidelity.

**FIGURE 1:**
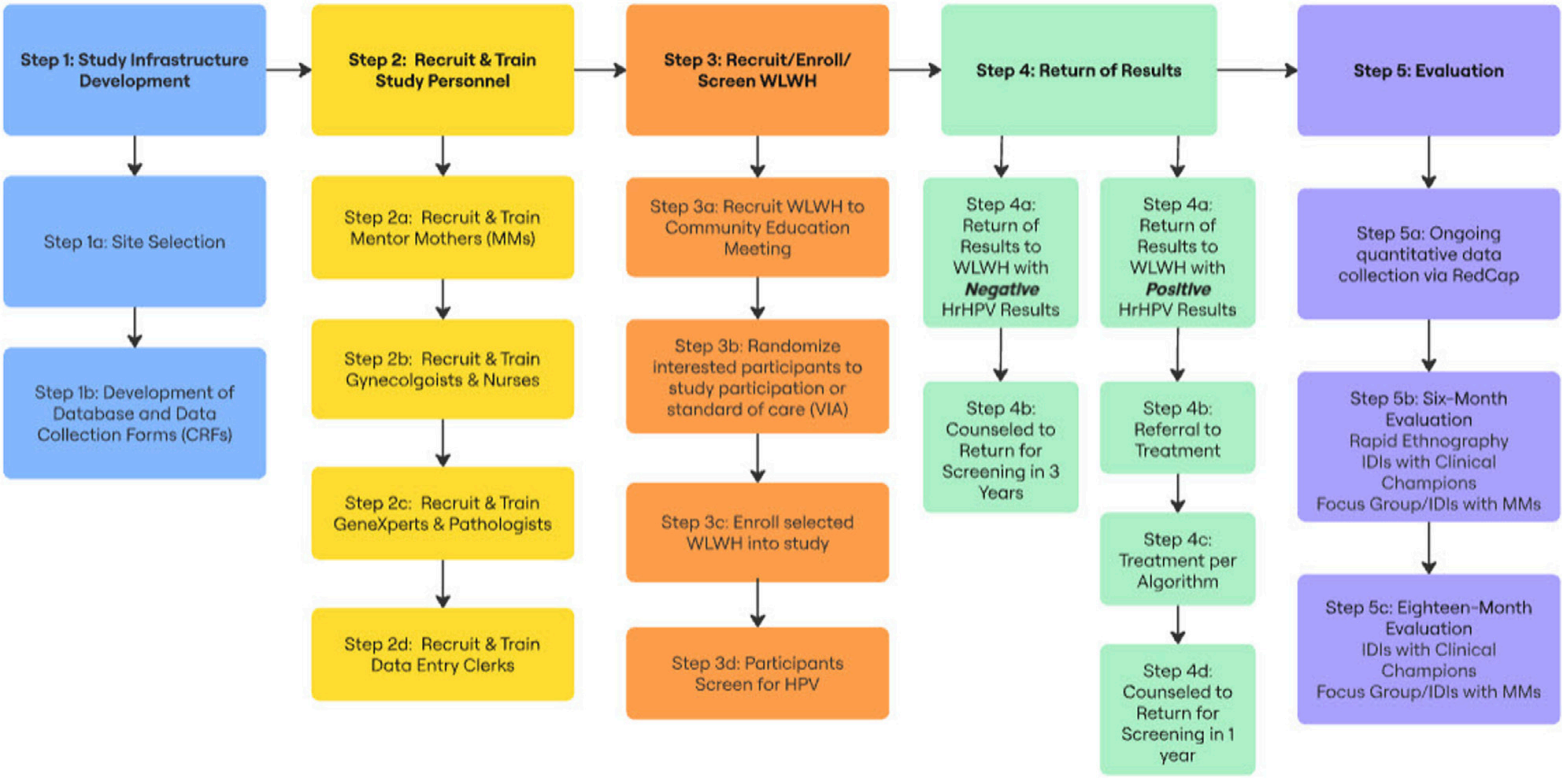
Aim 2 implementation flow chart (CHESS: Community, Home-based Education, Screening Services, Nigeria, 2025).

The details of Aim 2 are provided below and summarized in Figure 1.

We will implement the adapted MoMent HIV+HCC program in 15 Nigerian clinical sites to recruit 30 MMs and 1500 WLWH for mid- and post-implementation assessments of reach, effectiveness, adoption, and fidelity. Implementation outcomes will be evaluated 6 months post-implementation with mixed methods. Key outcomes include reach and effectiveness, as indicated by eligible women touched by the HIV+HCC intervention, uptake of screening, women with high-risk HPV (hrHPV) 16/18/45/other genotypes treated, persistent or recurrent hrHPV post-treatment, and correlation of disease persistence with CD4 count and HIV viral load. We will measure program adoption outcomes that include training of MMs, WLWH who return a SC sample, and participating clinics’ integration of MoMent HIV+HCC into routine practice. We will gauge implementation fidelity as indicated by peer leaders’ progress reports. We will also measure system barriers and added cost of CC screenings.

#### Site Selection

Fifteen PEPFAR-supported clinics across 8 Nigerian states were selected based on geographic diversity (including urbanicity), level of care, ownership, and patient volume, to represent the diversity of care settings in Nigeria. Each site aims to recruit 100 WLWH aged 25–50.

#### MoMent HIV+HCC Adapted Intervention

The MoMent HIV+HCC adapted intervention will consist primarily of MMs supporting WLWH in testing for hrHPV. MMs will be responsible for recruiting and enrolling WLWH into the study, educating them about HPV and SC, transporting samples to the clinic/laboratory, returning testing results and facilitating treatment for WLWH who need follow up care. Details are described below.

#### Clinical Follow Up and End Points

hrHPV negative WLWH will be counselled to repeat the HPV test in 3 years and counselled that a negative result does not indicate future protection from HPV.

All WLWH who test positive for hrHPV 16/18/45/other will undergo visual inspection with acetic acid (VIA). Patients with acetowhite lesions after application of 3%–5% acetic acid per VIA protocol [31] will have these areas biopsied for diagnosis. Those who do not demonstrate acetowhite lesions on VIA (VIA-negative) will have 4 quadrant biopsies on the cervix along the squamocolumnar junction. For patients where the squamocolumnar junction is not fully visualized secondary to being in the endocervical canal, an endocervical curettage will be collected. If the patient qualifies for thermal ablation treatment, she will receive same-day treatment. However, if the lesion is ineligible for thermal ablation, the patient will be referred for excisional procedure for diagnosis and treatment. VIA, biopsies, and treatment will be performed by the clinic gynaecologist and biopsies will be sent to the state pathology laboratory.

All hrHPV WLWH treated per the protocol will repeat a self-test for HPV at 1 and 2 years from the time of treatment to determine clearance/persistence of hrHPV infection. If the WLWH is positive for hrHPV at 1- or 2-year post treatment, they will undergo VIA and biopsies and, based on the gynaecologist’s recommendation, repeat thermal ablation if eligible, or excisional treatment.

All test and treatment outcomes will be correlated with the CD4 count and viral load of the participants. As will be specified in consent forms, all left over aliquots from all cases of positive hrHPV, pathology blocks, and slides will be archived for future relevant studies. The HPV SC samples will also be available for full HPV genotyping.

#### Recruitment of Mentor Mothers (MM)

We will use purposive sampling techniques to select 30 HIV+HCC MMs from among existing MMs in the 15 selected facilities. Two MMs will work at each site and share responsibility for 100 WLWH. MMs will provide counselling and support, encourage clients to uptake the CC screening, facilitate the screening process, return HPV results, and provide support services for necessary treatment. Selected MMs will demonstrate: (1) 4+ years’ experience as an MM, (2) sustained viral suppression, (3) good adherence to drugs and clinic appointments, (4) history of advocating for people living with HIV, (5) history of returning patient logs, and (6) experience with CC screening.

#### Training MMs for the Adapted HIV+ HCC Program

Two MMs, a senior MM and a junior MM, as defined by length of time working as an MM at that clinic, were selected for participation in the CHESS study. The senior MMs were invited to a two-day, interactive training. Training included an overview of the CHESS project and information on HPV, CC screening, and project-specific MM responsibilities. MMs conducted their own HPV SC test using the Evalyn Brush tool [32] (HPV results were returned privately to each participating MM). MMs completed pre- and post-training assessments and were required to demonstrate proficiency (>80% correct responses) across all topics or require retraining.

Senior MMs who participated in the in-person training returned to their clinic and trained the junior MMs using study-provided workbooks and materials. Further, an online refresher training was held 3 weeks prior to the start of study implementation to review all study elements with all MMs and clinic site managers.

#### Training Clinicians for the Adapted HIV+HCC Program

We conducted a clinical training for gynaecologists and nurses who will treat WLWH who have hrHPV results. Clinicians were trained in performing cervical biopsies for documentation of histological grade of disease and conducting thermal ablation and LEEP. Trainees also worked through case presentations to assist with decision making during colposcopy and treatment.

Clinicians completed pre- and post-training surveys to assess skill improvement.

#### Training GeneXperts and Pathologists

Our study addresses critical diagnostic challenges related to p-16 testing, a key marker for HPV-associated cervical dysplasia. Our pathology team, with two Nigerians and one American pathologist, identified significant gaps in their ability to perform p-16 testing due to lack of essential supplies, such as the P-16 primary antibody and detection kits. Nigerian pathologists also do not have access to digital microscopes, which are crucial for remote consultations and quality control. To address these limitations, the study procured digital smartphone microscope adaptors, which will enhance diagnostic accuracy and facilitate second reviews by pathologists for quality assurance.

As part of our efforts to standardize diagnostic procedures, we convened a meeting with Nigerian pathologists, virologists, and an Emory pathologist to review the study schema, particularly the 4-quadrant biopsy collection process. This review was essential to ensure consistent collection techniques and reliable diagnostic outcomes. We also examined the indications for p-16 staining, ensuring it is appropriately applied to cases where HPV-related dysplasia is suspected, organized training on the use of GeneXpert for HPV testing and collaboratively worked on the distribution of HPV testing cartridges and transport medium. Furthermore, we standardized terminology used on treatment evaluation forms for vaginal biopsies, ensuring that all sites are aligned in documenting biopsy results and treatment evaluations accurately.

We established quarterly quality assurance conferences to maintain diagnostic consistency, especially in the interpretation of various levels of dysplasia. Our discussions with the virologists also focused on refining protocols for sample collection, storage, shipping, and necessary reagents to ensure sample integrity. These coordinated efforts aim to enhance local diagnostic capacity while adhering to rigorous quality control standards.

#### Recruitment of WLWH

We will recruit 1500 consenting WLWH from 15 healthcare facilities (100 per facility) across eight states over a period of 18 months. A sampling frame of WLWH will be generated using a report run by the clinic staff of eligible women currently receiving care. Eligible WLWH will be contacted via telephone by the MM who will provide information about the study process. WLWH who are interested in participating will be invited to a community education meeting. Upon arrival at the community meeting, the MM will educate about HPV screening, enroll interested participants into the study and facilitate logistics for the self-screening process in a private location at the meeting site.

Eligibility inclusion criteria include:

- Female gender
- Diagnosed with HIV and receiving care at study clinic
- Ages 25–50y
- Able to consent to the study

The decision to screen WLHIV age 25–50 years is based on WHO recommendation which recommends HPV screening from age 25 years up to 65 years when there are tools to manage such women. However, we chose to limit our screening to upper age of 50 years because neither VIA nor ablative treatment, which are our proposed triaging test and first line treatments respectively, are suitable for women in whom the transformation zone is not visible which is typical after menopause. WLWH will not be eligible if they are pregnant, have had a hysterectomy or are not able/willing to perform vaginal self-collection.

A sample size of 1500 WLWH was determined after considering a design effect of 1.2 (to adjust for cluster sampling) and then incorporating a dropout rate of 20%. A sample size of 1500 has 89% power to detect an absolute difference in proportions of 0.04 using a two -sided exact test with a significance level of 0.05. This calculation assumes that the population proportion under the null hypothesis (P0) is 0.79. This is based on previous research that identified the proportion of women that are willing to screen for CC [33, 34].

Each week, MMs will call eligible patients and invite them to a culturally appropriate education session on HPV screening and SC. The MM will also introduce the CHESS study and take names for women who are interested in enrolling.

The Site Coordinator will receive the list of interested women from the MM and, given resource limitations, will use a random number generator to select 10 women/month. The MM will contact the selected participants and arrange to meet them in the community, where they will consent and enroll the WLWH. Women not randomly selected will be offered alternative screening through standard of care (VIA). Upon enrollment, the participant will self-collect an HPV sample in a private space using the Evalyn Brush [32], aided by pictorial brochures and videos developed in English and the three most common Nigerian languages. MMs will transport the labelled samples securely to a laboratory for testing. The Evalyn Brush, whose accuracy is similar to clinician-collected samples [35] may be stored dry in the container it comes in at room temperature and remains stable up to 6 months. If a private collection space is not available, the MM and participant will make a plan for the return of the sample within 10 days of collection. HPV testing will be done on aliquots of samples using GeneXpert HPV platform to determine sample HPV positivity and intervention decisions. Laboratory processing of cervicovaginal samples entails pipetting 1 mL of each HPV sample into the GeneXpert HPV cartridge and then slotting the cartridge into the GeneXpert machine for processing.

The MM will be responsible for returning test results to participants. For results indicating no presence of hrHPV, the MM will counsel the participant on the meaning of the results and encourage her to retest in 3 years. For results indicating the presence of hrHPV, the MM will work with the nurse at her clinic to facilitate further evaluation and treatment by a gynaecologist.

#### Development of Data Management Systems and Tools

Study data will be managed in REDCap. Access to the password-protected database will be restricted to approved study team members. Data will be stored on institutionally certified, password-protected, and encrypted tablets, laptops, and external drives. Only approved study staff will receive data via secure file transfer platforms.

The study team collaboratively designed Case Report Forms (CRFs) for easy completion and data entry. Questions were tailored to support analysis and quality control. MMs will complete CRFs at various CHESS study stages, and clinical personnel will complete during or immediately after exams. All CRFs will be submitted to clinic-based data clerks within 48 h. Electronic data entry screens will replicate the CRFs. Site clinicians and data managers were trained in CRF completion and REDCap data entry during a virtual pre-study session with refreshers offered. Missing or anomalous data will be sent back to sites for clarification or resolution.

#### Evaluation Using Reach, Effectiveness, Adoption, Implementation, and Maintenance (RE-AIM)

RE-AIM is a framework for planning and evaluating that assesses 5 dimensions: reach, efficacy, adoption, implementation, and maintenance [29, 30]. These dimensions occur at multiple levels including individual, clinic or organization and community and interact to determine health impact of the program. As shown in Table 1, we address relevant RE-AIM constructs for the study sample overall and by state.

Guided by the RE-AIM framework, we will use a convergent mixed-methods approach to implement a six-month, post-implementation assessment of reach, adoption, implementation, and fidelity. Quantitative and qualitive data will be collected in parallel and analyzed separately, then integrated during interpretation. Quantitative measures include numbers of women contacted, screened, and treated, numbers of MMs trained, and numbers of clinics that reach the optimal ratio (1:50) of MMs to WLWH. We chose this ratio on data from clinics in the six participating Nigerian states with input from experts in the field. The 1:50 ratio balances the needs of WLWH and the resource constraints faced by clinics/MM programs providing care. Data sources include site observations, clinical records, reports, training logs, and sample analysis. Quantitative outcome measures and data such as socio demographic/socioeconomic characteristics of participants will be analyzed using descriptive statistics and compared across states using the chi-squared test at the 5% level of significance.

To measure system flow, we will conduct rapid ethnography at a subset of 6 clinics (one per state) to assess daily clinical processes, recording observations in field notes. We will also follow 80 WLWH (10 per clinic) through the process, timing each step and noting barriers. Additionally, we will conduct virtual in-depth interviews (IDIs) with a clinical champion at each clinic (n = 15) and two focus groups (total n = 10–12) with HIV+HCC MMs to identify implementation barriers and facilitators. We will focus on MMs, who understand both systemic issues and the individual experiences of WLWH. Clinic champions will be asked to gather input from other staff before the IDIs to inform responses. Stakeholders will be informed they will participate in follow-up qualitative data collection at 18 months (Aim 3). IDIs and focus groups will be audio-recorded and transcribed, with a team member taking notes. Preliminary thematic analysis will be conducted to identify key barriers and facilitators to inform project adjustments.

Finally, we will capture project costs of adding the HCC components into the MoMent program as considerations for maintenance and sustainability. Measures will include costs for labor (clinical and laboratory staff and MMs), training and education, and clinical materials and supplies related to screening (e.g., Evalyn brush, GeneXpert Cartridge) and diagnosis (VIA consumables). Integrated findings will inform program refinement and support recommendations for scale-up and sustainability.

Aim 3 Conduct post-implementation process evaluation of barriers and enablers to program maintenance.

In the maintenance phase, we will evaluate how the intervention has become routine practice at 18 months post-implementation. We will again collect data from the same stakeholders identified for Aim 2. Stakeholders’ viewpoints and processes related to the RE-AIM construct of “maintenance” will be assessed via quantitative measures (Table 1), structured interviews (n = 15), focus groups (n = 10–12), and numbers of WLWH screened/treated during the maintenance period. Stakeholders will also identify barriers to maintenance, resources available to support and capacity to scale the intervention across Nigeria.

### Study Timelines

Broadly, our timeline follows the three study aims (Table 2): Aim 1 consists of the SD conferences and intervention adaptation which occurred in the first 18 months of the grant. Aim 2, which covers the implementation of the adapted intervention and six-month post intervention assessment, will happen in years 3–4. Aim 3, which includes the 18-month maintenance evaluation, will happen in years 4-5.

**TABLE 2:** Study implementation timeline across project years. (CHESS: Community, Home-based Education, Screening Services, Protocol, Nigeria, 2022–2025).

Aim/Activity	Year 1		Year 2		Year 3		Year 4		Year 5	
**Aim 1:** Stakeholder Deliberations										
Development of SD process & data collection materials	X									
Recruitment of participants	X									
Hold SD conferences & analyze data	X	X								
Adapt HIV+HCC intervention		X	X							
Train clinic staff & peer support leaders to implement intervention		X	X							
**Aim 2:** Intervention Implementation & Evaluation										
Development of aim 2 data collection tools		X	X							
Implement HIV+HCC intervention				X	X	X	X	X		
Six-month post-implementation assessment data collection					X	X				
Six-month post-implementation assessment analysis						X				
Make necessary adaptations to HIV+ HCC intervention						X				
**Aim 3:** Post-Implementation Evaluation of Maintenance										
Development of aim 3 data collection tools							X			
18-month maintenance assessment data collection								X		
18-month maintenance assessment analysis								X	X	
Stakeholder dissemination									X	
Writing, presentations, publications									X	X

## Results

Project results will be shared with researchers, practitioners, and policymakers. Aggregate findings will be disseminated to MMs, the project team, healthcare providers, hospital administrators, NGO stakeholders, academics, government officials, media, and funders. Channels will include MM trainings, seminars, conferences, policy briefs, letters to the editor, peer-reviewed journals, social media, and career platforms. Dissemination will address implementation processes, facilitators, barriers, strategies, and outcomes to enhance cervical cancer screening uptake and scale-up for women living with HIV, leveraging MM expertise.

A dissemination team—including project members, MMs, WLWH, and other stakeholders—will share information through meetings, digital platforms, and media. Efforts will be tailored by audience, considering language, interests, and purpose. Policymakers at local, state, and federal levels will receive policy briefs to support scaling cervical cancer screening in Nigeria. Principal investigators will evaluate dissemination to assess success and impact. The goal is to implement locally appropriate strategies that improve care quality and outcomes for WLWH in an LMIC setting.

### Limitations

This study is conducted in Nigeria and may have limited generalizability to other LMICs with different health system structures, sociocultural norms, and policy environments. Implementation challenges such as resource limitations, variability in MM training, and infrastructure constraints may affect program scalability and sustainability. Additionally, the reliance on self-reported data and clinic records may introduce reporting bias or incomplete outcome capture.

## Discussion

Nigeria has planned future resource allocation toward CC control and treatment [36], which were historically covered by non-governmental organization funds. This expansion of federal funds will allow for significantly expanded CC screening and treatment of WLWH nationwide.

Within this context and building upon the success of peer-support programs such as MoMent, our study will show the best approach to delivering a CC self-screening and treatment program in Nigeria for WLWH. This study also has the potential to expand equitable access to CC screening in LMICs beyond Nigeria.
